# From playing styles to game model: deconstructing the paradigm through analysts' perspective

**DOI:** 10.3389/fspor.2026.1825139

**Published:** 2026-07-24

**Authors:** Adrián Martín-Castellanos, Diego Muriarte Solana, Claudia Portillo Martín, Enrique Alonso-Pérez-Chao, Daniel Mon-López, Javier Coterón

**Affiliations:** 1 Departamento de Deportes, Facultad de Ciencias de la Actividad Física y del Deporte (INEF), Universidad Politécnica de Madrid, Madrid, Spain; 2 Facultad de Ciencias Biomédicas y de la Salud, Universidad Alfonso X El Sabio, Madrid, Spain; 3 Department of Real Madrid Graduate School, Faculty of Medicine, Health and Sports, Universidad Europea de Madrid, Madrid, Spain; 4 Departamento de Ciencias Sociales de la Actividad Física, del Deporte y del Ocio, Facultad de Ciencias de la Actividad Física y del Deporte (INEF), Universidad Politécnica de Madrid, Madrid, Spain

**Keywords:** fluid styles, football, qualitative research, rigid styles, tactical behaviour

## Abstract

**Introduction:**

Understanding how teams organise their tactical behaviour remains a central issue in football performance analysis. Although the concept of playing style has been widely discussed in the literature, its definition and practical interpretation remain inconsistent across both research and professional practice. Therefore, the aim of this study was to explore how professional performance analysts conceptualise and interpret playing styles in elite football.

**Methods:**

A qualitative and interpretative design was employed using semi-structured interviews with eighteen professional performance analysts from the two top competitive levels within Spanish football leagues, as well as national teams. The data were analysed using reflexive thematic analysis, allowing the identification of recurring patterns and shared interpretations regarding the conceptualisation and practical use of playing styles.

**Results:**

The results indicate that analysts perceive playing styles as part of a broader behavioural construct that defines general tendencies across teams, while the game model represents the contextual and team-specific application of these tendencies. Possession-based, direct and transitional play emerged as the predominant offensive styles, whereas defensive styles remain less conceptually defined. The findings also highlight the influence of contextual, competitive and individual factors, including the coach's philosophy, players' characteristics, level of competition and the tactical approach of opponents. These elements determine the degree of rigidity or fluidity expressed within each game model.

**Discussion:**

The study proposes a theoretical framework that integrates offensive and defensive dimensions of play and situates rigid and fluid styles within the team game model. This framework enhances conceptual clarity and provides analytical value for future research and applied performance analysis in modern football.

## 1 Introduction

The tactical analyst is a highly demanded role in professional football staff (1), due to his support to the technical staff by analysing the opponents through video, studying their behaviour, patterns, and movements, and providing strategic proposals. Moreover, analysts often contribute to examining their own team's performance at different stages of the competition. To this end, two of the principal skills necessary for this position are understanding the game and a clear observation methodology (2).

Sarmento et al. (3) emphasised that collective system behaviours represent a key area of analysis in team sports. Within this framework, the behaviour adopted by teams in offensive and defensive phases to achieve their objectives in a match can be defined as their playing style (4). Playing style seems to develop a crucial role in predictions of opportunity to score, being identified as the cause and solution for different scoring models (2). Nevertheless, the literature reveals diverse conceptualizations and methodological approaches to defining and analysing playing styles (5–7).

In an effort to identify playing styles, Fernández-Navarro (8) conducted a study in which professional coaches recognized 14 distinct styles, based on the phases of play outlined by Hewitt et al. (9). These included direct play, possession, crossing, inner play, playing in the wings, vertical, high pressure, middle block, retreat, individual marking, pressure after losing the ball, counterattack, fast attack after regaining and set piece. Other quantitative approaches have sought to identify playing styles through data analysis and multivariate techniques (10). While such methods offer insights into style variability, playing style remains a complex and multidimensional concept to operationalize (10, 11). Therefore, incorporating more qualitative analyses of real-world processes could enhance the scientific development of this field (12).

Due to the diversity of methodological approaches, classifications of playing styles may sometimes appear overly granular or lack contextual specificity. Moreover, literature does not always provide clear or consistent definitions for each identified style. As the volume of publications on this topic continues to grow, understanding the role and interpretation of playing style becomes increasingly relevant. In this context, the ambiguity also extends to the distinction between playing style and game model. While playing style refers to the general patterns of behaviour observed in different teams, the game model corresponds to the specific application of those behaviours by each team (13). However, both concepts are frequently used indistinctly in the literature and in professional practice, which reduces their conceptual clarity and analytical utility.

Consequently, it is crucial to incorporate the perspectives of expert analysts, who can contribute to the identification and categorization of these styles and the teams that employ them. In line with this, the present study aims to analyse and characterise the playing styles adopted by teams through interviews with professional analysts.

## 2 Material and methods

This study adopts an exploratory and descriptive design to gain a more in-depth understanding of a theme through the perspective of qualified professionals based on their personal experience in top-level football.

### 2.1 Participants

The participants consisted of 18 male professional analysts (36.27 ± 7.86 years; 5.83 ± 3.16 years of experience in professional football). The inclusion criteria for participation in this research were (i) being part of a men's football coaching staff in their professional categories (La Liga Santander or LaLiga Smartbank, corresponding to the first and second divisions of professional football in Spain) or (ii) be employed within a national team coaching structure.

Participants were initially contacted through professional social networks, including WhatsApp, Twitter, and LinkedIn. Following the first set of interviews, a snowball sampling strategy was implemented: participants were invited to refer colleagues who met the inclusion criteria, thereby expanding access to relevant professionals.

All procedures were conducted in accordance with the ethical guidelines established by the American Psychological Association (14) regarding informed consent, confidentiality, and the anonymity of responses. A record of verbal informed consent was obtained from all participants, and pseudonyms were used in reporting results to ensure confidentiality. The study received ethical approval from the Ethics Committee of the Universidad Politécnica de Madrid, Spain.

The sample size was considered appropriate in line with the concept of information power, as the study involved a relatively specific and experienced group of participants, a focused research aim, and in-depth qualitative data obtained through semi-structured interviews (15).

### 2.2 Data collection

A semi-structured interview was specifically developed for this study to explore analysts' perspectives on playing styles and their professional practice. Prior to its implementation, the interview protocol underwent a thorough review by two graduate researchers in Sport Sciences and two PhD experts in the field, specialised in football and qualitative research. Based on their feedback, necessary revisions were made to improve the clarity, relevance, and coherence of the questions.

The interview was organised around two main thematic areas: (i) the analysis process adopted by performance analysts, and (ii) the conceptualisation and practical interpretation of playing styles. Open-ended questions were used to explore participants' experiences and perspectives in depth. The interview guide is provided as Supplementary Material (Annex 1).

Subsequently, a pilot interview was conducted with an experienced professional football analyst. Upon completion, the analyst was invited to provide feedback regarding potential improvements. This feedback was incorporated into the final version of the interview.

The interviewees provided informed consent for the analysis and use of the information they shared. The average duration of the semi-structured interviews was 43.77 ± 12.67 min. The interviews were conducted and recorded via Zoom for subsequent analysis and then transcribed for thematic analysis. The NVivo software (Version 1.6.2) for Windows (QSR International, Burlington, MA, USA) was used to analyse the data. All collected data were securely stored in an anonymous and encrypted format within the university database of the principal investigator (PI). Access to any personally identifying information was restricted solely to the PI, ensuring both confidentiality and data protection. Data storage and handling procedures adhered strictly to the requirements of the Spanish General Data Protection Regulation requirements.

### 2.3 Data analysis

Data were analysed using reflexive thematic analysis (16–18). This approach was selected for its theoretical flexibility and its capacity to capture participants' meanings and experiences in depth. Trustworthiness was addressed throughout the analytical process in line with the criteria proposed by Nowell et al. (19).

Following the transcription of the audio recordings into Word® documents (Microsoft Corporation, Redmond, WA, USA), the transcripts were read and re-read to achieve familiarisation with the data and to identify units of analysis. During this phase, preliminary reflections were noted, and the data were organised systematically to ensure transparency throughout the analytical process, following the general principles outlined by Mayring (20).

Subsequently, initial coding was conducted inductively through an open coding approach, resulting in a total of 652 coded units. Codes were generated from the data and subsequently organised according to emerging patterns that were representative of participants' perspectives, following principles of internal consistency and coherence. As the analysis progressed, codes were iteratively reviewed and compared across the dataset to support the identification of patterns of shared meaning and the development of themes. The research team held regular meetings to discuss interpretations and refine the analytical process, supporting a reflexive engagement with the data.

Provisional themes were reviewed iteratively to assess their coherence and their alignment with the dataset. Themes were then defined and refined, and a final analytical narrative was developed, linking the themes to the research question and supported by illustrative data extracts. Additionally, and in line with previous qualitative research (19), researcher triangulation was considered as a complementary criterion to support the analytical process through the inclusion of multiple perspectives.

## 3 Results

The analysis of the interview data led to the identification of several key themes, which are presented in the following sections. These themes reflect the most significant findings that emerged from the participants' responses. Each theme is supported by illustrative quotes to enhance the transparency and depth of the analysis.

### 3.1 Blurring the lines: playing style or game model?

While the concept of playing styles is frequently referenced in football discourse, its practical relevance within professional analysis may be questioned. Several participants in this study expressed doubts regarding the operational utility of clearly defined playing styles, suggesting that their application may be more theoretical than functional. Within this framework, some analysts expressed difficulty in articulating consistent or precise definitions of playing style, and the term was used in ways that reveal a certain degree of conceptual ambiguity.

“The game doesn't allow you to create a style, because, as I said, this is not cinema. Here it's a competition in which I have a rival in front of me and I have to adapt … So, the game, depending on all that, will start to send me things and I have to satisfy them.”—Analyst 11

“Well, a playing style would be a set of behaviours performed by the players, we could say, based on the coach's ideas, right? I think that's what most closely resembles what we mean by a playing style.”—Analyst 4

“Each team has its own style, even if you can pigeonhole it into one of them. In the end everyone has their, their style, and every team is different. In the end we can name a style of play with more possession. A style of play with more direct play, more defensive … but we don't do ‘this team plays like this', and this team plays like that  … we describe it but we don't categorise it.”—Analyst 5

This may be influenced by the way the concept of playing style is approached, as analysts often focus on the opponent's patterns of play (game model). This suggests that the notion of playing style may not consistently defined. Although some of the definitions provided by participants aligned with concepts found in the existing literature, this consistency was not observed across all responses.

“I define style and model of play, which is not the same thing. So, I separate the style I'm going to wear, you and I don't dress in a certain way. I wear a suit, and you wear pop. That's your style. Pop style but classic style. And the model is, within the style, if I wear a tie, if I don't wear a tie, if I wear a bow tie, if you wear a tracksuit, if you wear trousers and its jeans, if that's what I define it as.”—Analyst 13

### 3.2 Two traditional but foundational playing styles … and a third contender

From an initial analytical perspective and given the limited emphasis placed on the concept of playing styles by analysts, it may be assumed that such inconsistencies have minimal practical relevance. Nevertheless, most analysts identified two primary playing styles: direct play and combinative (also referred to as associative or possession-based) play.

“In Spanish football, I do notice that we seem to be divided into two main tendencies in terms of playing styles: on the one hand, the associative style of play, and on the other, teams that follow a more direct approach.”—Analyst 9

“I am not in favour of categorising styles of play. It is simply the way I see it. However, it is clear that there are two distinctly different models: direct play and combinative play.”—Analyst 7

The direct style of play is generally characterised by a deliberate emphasis on rapid ball progression and verticality. This approach often involves the use of long passes, aerial challenges, and the exploitation of early positional imbalances. Teams that adopt this style typically seek to move the ball into the opponent's half as swiftly as possible, aiming to bypass intermediate build-up phases and capitalise on second-ball situations.

“For me, it would be a playing style based on the idea of initiating play and trying to progress into the opponent's half through combination play. Alternatively, there is an approach that involves forcing the ball forward, either through physical means or by engaging in duels, aiming to win these duels.”—Analyst 2

On the other hand, the possession-based style is typically defined by a clear intention to initiate play from the back, maintain control of the ball, and progress methodically through coordinated movements and short passing sequences. Its ultimate aim is to destabilise the opposition by gradually breaking through defensive lines. This approach is often associated with teams that aim to dominate possession and take a leading role in the game, particularly during organised attacking phases. Specific teams can even be identified by different analysts as clear examples of both styles:

“There are teams such as Girona or Las Palmas that you know will initiate almost every action from the back. Real Sociedad B, a reserve team, also tries to play that way. But then there are other teams, like Lugo or Amorebieta, that you know will play long.”—Analyst 2

“Teams like Real Sociedad, FC Barcelona, Villarreal or Celta de Vigo represent more combinative or associative styles of play. They aim to draw the opponent in, create space, and generate situations in which the opposing team becomes disoriented. In contrast, other styles are somewhat more direct, such as the one we used or the one played by Eibar, which are generally less risky in terms of build-up play.”—Analyst 9

However, a minority of analysts referred to what may be described as a third style, primarily characterised by offensive transitions, which may be closely associated with counterattacking strategies. This style differs in that it often originates from a fundamentally defensive posture. Teams employing this approach tend to remain compact in a low or medium block, inviting pressure in order to create space behind the opponent's defensive line. Once possession is regained, they prioritise vertical progression at speed, seeking to exploit disorganisation through quick, direct attacks. This reactive logic contrasts with the proactive intention of both possession-based and direct attacking models.

“In terms of playing styles, I imagine it comes down to those who aim to be protagonists with the ball, particularly in the phase of organized attack, and those who rely more on a compact defensive block in order to attack through transitions … . And then there are transitional teams, such as Valladolid, for instance. A team that, when counterattacking, would send ten players into the box. A very brave team in high pressing, whose main intention was to press, recover, and break forward. Press, recover, and break.”—Analyst 3

“Whether it is a combinative attack or direct play, it depends. Some teams prefer to defend and focus on transitional phases. They wait, attempt to draw the opponent in, and then look to exploit the space behind the defensive line.”—Analyst 6

### 3.3 Attack defines styles … but does defence follow?

From the analysts' perspective, offensive behaviour appears to be the primary reference point when identifying playing styles. Nevertheless, numerous references were also made to the way teams organise their defensive phase, with several analysts suggesting that a potential relationship may exist between the attacking approach adopted by a team and the defensive structure it employs.

“Whenever we talk about playing styles, it always seems to refer to when we are in possession. But there are also moments when we do not have the ball, and my team might spend more time without it and still exhibit a particular playing style.”—Analyst 9

“Most of those offensive team prototypes are almost always closely linked to a specific defensive approach.”—Analyst 17

However, several analysts pointed out that certain teams either prioritise or are conditioned by specific phases of play, particularly the defensive phase. Nevertheless, they generally did not provide explicit definitions or systematic characterisations of these phases, often limiting their observations to aspects such as the height of the defensive line.

“Speaking in terms of ball possession, such teams also tend to operate, during the offensive phase, with a lower block. Or rather, not necessarily a lower block, but not exclusively with a high one.”—Analyst 1

“For example, Atlético de Madrid under Simeone a couple of seasons ago defended with what would be considered a medium block.”—Analyst 18

### 3.4 Rigid or fluid playing styles?

In characterising playing styles, analysts noted that certain teams display a rigid and clearly defined tactical identity, shaped by specific tactical elements that remain constant and are closely aligned with the overarching philosophy of the coach or the club.

“ … for example, Girona. You know that if you're going to put pressure on them up top, even if you go to man with Michel, they're practically going to try to come out and play. So, we categorise them as teams initiating play. Elaborate, yes or yes. On the other hand, teams like Amorebieta. You know that even if you don't go, you're going to be thrown long, with players who are good at that type of play … There are teams that have a style more based on what they have in front of them and there are other teams that have a rigid style. And many times, they die with it. 90% of the time they die with an idea that the coach has.”—Analyst 2

This may be determined by the coach's idea of the game model since the identification of the teams is accompanied by the name of the coach, making an allegory of the team's model of play. Analysts also refer to teams such as Michel's Girona or García Pimienta's Las Palmas when asked about similar teams (both in the second division at the time). However, some teams adopt an opposing approach, structuring their game plan primarily around the characteristics of their opponents and relying on the notion that the conditions and demands of the game will be shaped by the opposition's behaviour.

“Atalanta adapted its playing approach significantly depending on the opponent's structure. If the opposing team played with a back five, they would use one strategy; if the opponent played with a back four, they would adopt a different one. For instance, Atalanta frequently sought to create one-vs.-one situations in order to recover possession high up the pitch or force mistakes, rarely defending in a very low block.”—Analyst 8

“I cannot do whatever I want, as I could in cinema or music, where there are no external demands. I would say that in the past, perhaps it was possible, but nowadays teams control nearly every phase of the game. I find it difficult to see otherwise, because I truly believe that modern teams press intensely, and often, when they are unable to push into a high defensive block, it is not by choice but because the opponent does not allow it. They cannot step up or break out from that situation.”—Analyst 11

These findings highlight a contrast between teams that maintain a clearly defined playing style and those that adopt a more adaptive approach depending on the opponent.

### 3.5 From idea to identity: the factors that determine the path from styles to models

Different scenarios can be identified that may influence or contextualise the development of both the playing style and the game model. In relation to the presence of rigid vs. fluid tactical approaches, distinctions appear to depend, at least in part, on the level of competition. Teams ranked higher in the league standings may be less likely to adapt their strategies to the opponent, possibly due to a more consolidated tactical identity or a greater ability to impose their game. By contrast, teams with lower classifications are often described as more responsive to external demands and therefore more inclined to adjust their approach according to the characteristics of each match. This seems to be the case in first-division teams, the tendency in second-division teams is usually closer to each other, so they tend to show greater adaptability.

“At (Real) Madrid you are the protagonist and you will always have an idea, an idea of play. I mean, in one way or another you will always have an idea, an idea of play. Whether the opponent is one or another, it does not matter, it is the same, and you always focus on your own team. You pay little attention to the rival, or at least we paid little attention. And it is true that when you are in teams with a lower profile, you must pay much more attention to what the opponent does or what you are going to do and maybe adapt to that … So you can see the contrast from one side to the other, in one case there is a session dedicated solely and exclusively to set pieces, defensive, offensive, whatever you want, and in top teams they simply do not have the time to stop (due to the busy fixture schedule) say here we are going to do this or here we are going to do that. That does not mean they do not do it, but that they do it in a different way, mainly in video.”—Analyst 6

“We noticed that, for example, during our first year in the first division, right after being promoted, in the first half of the season we managed to maintain, so to speak, the same essence and playing style we had in the second division. However, as the season went on, the level of the competition itself made it clear that we also had to adapt to that higher category, which is completely different from the second division.”—Analyst 9

One of the most frequently mentioned factors is the quality and profile of the players available within the squad. The technical, tactical, and physical attributes players may determine the extent to which certain styles are operationalised and translated into coherent game models. In this sense, player characteristics can act as both enablers and constraints, influencing not only the choice of tactical principles but also the degree of flexibility with which teams can adapt to different scenarios.

“The team's playing style is obviously defined by the coach. But the style is shaped by both the coaches and the players.”—Analyst 8

“I think styles are set by the players, okay? Talking about the style being combinative and associative play is because the profile of the footballer that he signs are of an associative profile.”—Analyst 14

“There are players who, for example, offer more depth than others. It's not the same if, for example—I don't know—let's take an example, Isco plays on the left wing for Madrid, or if Vinicius plays, okay?”—Analyst 13

Another factor that may influence playing styles involves contextual conditions, encompassing cultural and historical aspects of the country or club, as well as the match location. Furthermore, the opponent's tactical approach may induce adjustments in a team's game model, thereby challenging the categorization of its playing style.

“It is also important to distinguish between victories achieved at home and those obtained away, since some teams perform much better when playing on their own ground. Their style of play at home often differs from the one they adopt when playing away, and all of this must also be taken into account.”—Analyst 8

“Maybe there are games where, because the opponent has a very defined way of playing and they are not going to change it, we will be the dominant side, but not because we are better than them, or because we have more talent, or because we are a team that believes in keeping possession, but because if you face Cervera's Cádiz you will dominate the game, since the scenario they present is that they will give you the ball, drop back, and wait for you. And that match scenario, that style of game, will have you as the dominant team in possession. On the other hand, if you face Barcelona, you will play a completely different style of game.”—Analyst 1

“Pep Guardiola, widely recognized as a master of positional play and the tiki-taka style, provides a clear example of tactical adaptability. In the UEFA Champions League tie against Real Madrid, when the opponent applied man-to-man pressing, goalkeeper Ederson resorted to long passes to exploit one-on-one situations, such as Sterling's matchup with the central defender. This illustrates that Guardiola did not abandon his playing philosophy but rather adapted its application to the specific demands imposed by the opponent.”—Analyst 2

Nevertheless, there appears to be a lack of consensus regarding the relationship between game formation, game models, and playing styles. While some perspectives view formations as structural frameworks that shape the model of play, others consider them adaptable mechanisms that may vary according to the team's game model or playing style.

“I believe that a more elaborated or combinative style of play is usually best supported by the 1-4-3-3 formation. On the other hand, a more direct style could perfectly fit within a 1-4-4-2 structure. In the end, with two forwards, one will always challenge for the aerial ball, keeping constant pressure on the opponent, and I believe that, in terms of field positioning, the team feels much more protected in that formation … And why would it be the 1-4-3-3? Perhaps because of the distribution of the players on the pitch.”—Analyst 6

“If I play with a back five, I will obviously create greater density in the central areas while leaving the flanks somewhat more exposed. That is implicit in having only one wing-back, for example. The 1-4-3-3, on the other hand, tends to favour a more associative structure due to the presence of natural triangles across the pitch. Meanwhile, if I want to play associatively with a 1-4-4-2, it will require a lot of movement and rotations, since that formation provides a more symmetrical distribution of space.”—Analyst 15

“Valverde's Barcelona played in a 1-4-4-2 formation and was practically a combinative team. Stepping outside the 4-4-2 is often frowned upon, yet Guardiola himself has used it many times. The 1-5-3-2, on the other hand, is generally associated with a fast-paced game, featuring quick vertical passes that end in wide areas. However, there are occasions when Guardiola, or Luis Enrique for instance, employ a 1-4-3-3, and some teams use the same 1-4-3-3 structure primarily for defensive purposes.”—Analyst 10

## 4 Discussion

This research aimed to analyse and characterise the playing styles adopted by teams through interviews with professional analysts. The findings of this study invite a critical reflection on the practical relevance and conceptual clarity of playing styles within the specific context of Spanish professional football.

A primary finding of this study concerns the notable divergence between theoretical definitions of playing style and their interpretation within professional practice. According to Fernández-Navarro (8), the traditional concept of playing style refers to predominant behavioural patterns that emerge from the interaction of players during different phases or moments of the game (for example, direct or possession-based styles of play could be associated with the organised attack phase). From an analytical perspective, the element that could be considered as key is that these patterns must be common to different teams to be considered part of a style of play, otherwise they would be part of a game model, belonging to a particular team (13), and may be influenced (21) by the coach's ideas, players' skills, club's culture, structure and objectives, and even the national culture (22).

The styles of play predominantly identified by the analysts appear to correspond to more traditional frameworks, such as possession-based and direct play (23, 24). The former emphasises ball circulation and may have been influenced by the Scottish passing game (25) whereas the latter relies on long passes, individual duels, and minimal build-up phases (25, 26). It should be noted that these styles have been recognised in previous research (10). Similarly, the style related to transitions, which could be considered comparable to counterattacking play, has also been identified in several studies (10, 27), and may represent a growing tendency within contemporary football (28). Nevertheless, current trends in European football tend to prioritise positional play and direct attacking approaches, with possession-based play often described as a dominant tendency in recent years (29).

Nonetheless, the analysts' perspective diverges substantially from that of the coaches, who identified up to fourteen distinct styles of play (8). This high degree of variability in classification may lead to interpretative inaccuracies, as quantitative research has not yet been able to determine the precise number of existing styles (7, 11). Part of this variability may also be related to the methodological approaches used in performance analysis. Techniques such as principal component analysis may be limited by the number of variables included, which can restrict their ability to fully capture playing styles (5, 30). Similarly, clustering and other pattern-based analyses may present limitations, particularly due to issues such as overfitting and their reduced applicability in applied contexts. From a qualitative perspective, some categories may overlap due to their conceptual definitions, making their identification more complex within professional football analysis (8). These considerations highlight the need for further research that combines practical relevance with methodological rigor and can provide a stronger contextual foundation to better define different playing styles.

In addition, this variability may also be influenced by the growing emphasis on the defensive phase of play, which seems to have been overlooked in previous analyses that have primarily focused on offensive aspects of the game. This imbalance may have biased the conceptual boundaries of the categories employed in such studies. Although the defensive orientation of play has been examined in recent years (4), a shared framework of understanding still appears to be lacking. Analysts generally approach the defensive phase according to the areas of the pitch where they intend their teams to defend, thereby defining different defensive blocks, a notion that can be contrasted with the current body of literature (30–32). These findings can be understood within approaches that view team behaviour as adaptive and context dependent (33). This analysis provides a promising direction for future research, allowing for the examination of the interactions between offensive and defensive behaviours and supporting the development of more holistic tactical profiles and stable patterns of play.

**Figure 1:**
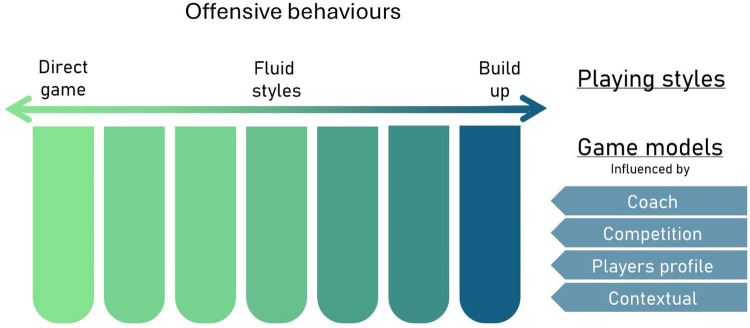
Example of offensive behaviours and their relationship with playing styles and game models, according to analysts' perspectives.

From this standpoint, the concept of style of play may serve to isolate certain categories and behavioural patterns of specific teams, thereby accounting for variability, yet without implying the exclusivity of one category over another. Furthermore, this variability, which stems from the natural tendency of teams to avoid adopting a single and repetitive style of play, could also account for the emergence of fluid styles that occupy an intermediate position between playing styles (Figure 1). These findings raise a relevant question regarding whether successful teams may rely more on the consolidation of a clearly defined playing style or on their ability to adapt effectively to different opponents (33). However, it appears that a multifactorial approach that considers various contextual and structural factors may better explain the development and implementation of a team's playing style and its subsequent translation into a coherent game model.

Moreover, when designing the match contexts and applying these playing styles through team game models, a range of factors may influence the tactical proposals and strategic approaches adopted by each team. Both the coach and the players appear to exert a substantial influence on how the game idea is translated into practice and on the selection of the playing style. Maintaining coherence between the characteristics of the coach and those of the players seems essential for the effective development and implementation of a specific style of play (34–36). This perspective aligns with recent approaches that conceptualise team behaviour as an emergent product of interactions between those factors (37, 38). In addition, both the level of competition (39) and the contextual environment in which a team performs can shape and determine its game model. For instance, FC Barcelona's playing philosophy appears to be closely linked to a possession-based style (40, 41). Further influential factors, such as the level or approach of the opponent (2) as well as by match location (42), are consistent with previous research, as they may alter the game model, although empirical evidence concerning these patterns of play remains relatively limited.

Regarding playing formation, inconsistencies emerge in literature due to divergent opinions among analysts. While this element of the game may exert an influence on factors such as physical performance (43–46), its direct impact on tactical identity remains less clear. Some authors suggest that formation may condition certain spatial and behavioural patterns within teams, particularly in relation to width, depth, and pressing height; however, the evidence supporting its impact on technical and tactical aspects appears to be comparatively limited. Consequently, playing formation should be considered as a flexible structural reference rather than a determinant of tactical identity.

**Figure 2:**
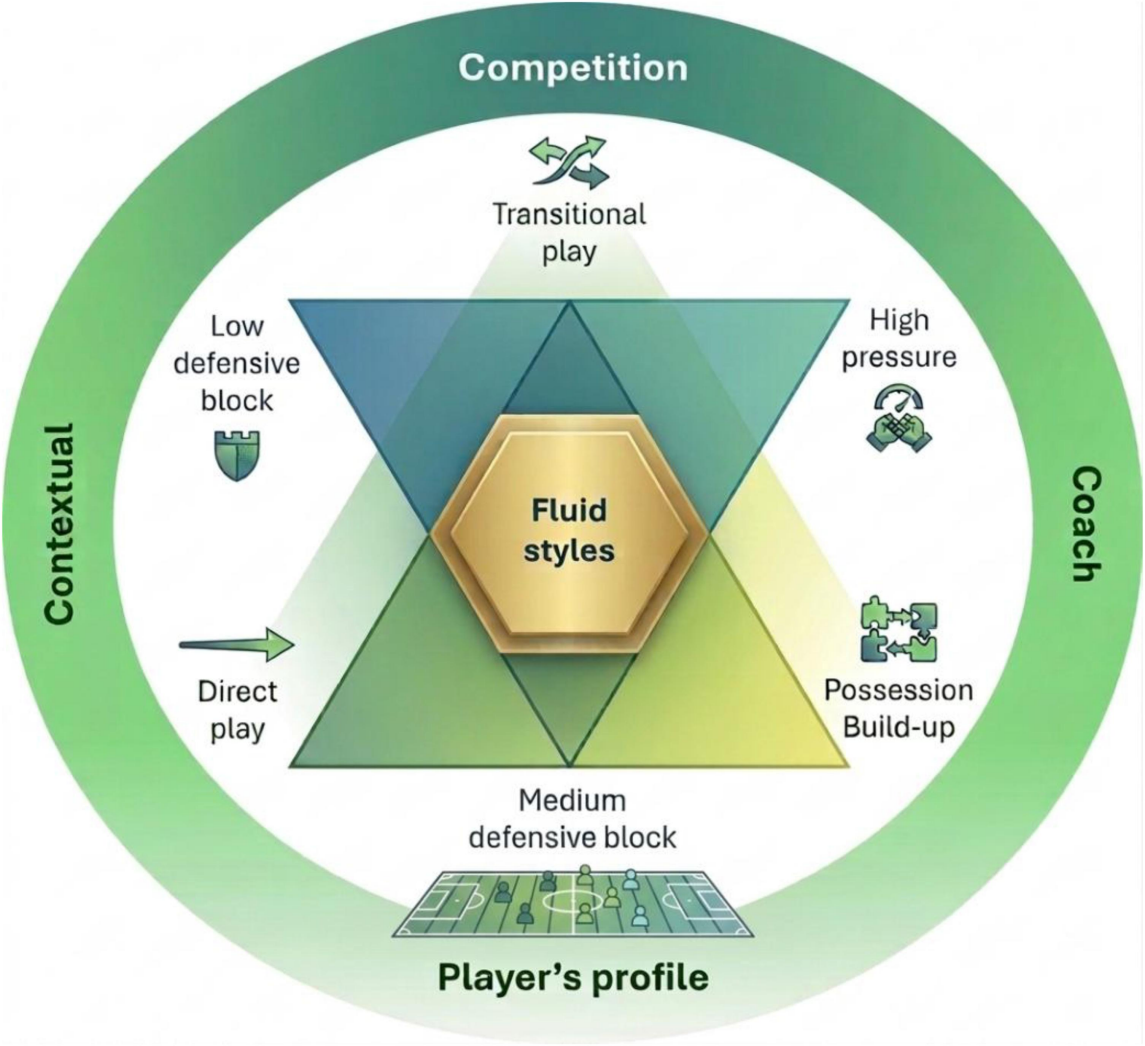
Theoretical framework of offensive and defensive playing styles, based on their degree of rigidity and the interaction factors influencing their applicability within team game models.

Finally, an interpretation of playing styles encompassing both offensive and defensive domains is proposed (Figure 2), considering their variability and including what can be described as fluid styles, understood as those that do not strictly fit into predefined categories. The applicability of these styles depends on the game models adopted by teams and is shaped by contextual factors such as the level of competition, the match environment, the coach's philosophy, and the players' characteristics.

However, the present study has several limitations that warrant consideration. First, the study sample is limited to a Spanish-speaking context, which may constrain the applicability of the findings to other cultural or competitive environments. In addition, the recruitment strategy may have introduced certain limitations. Snowball sampling is a valid and widely used recruitment technique, particularly appropriate for accessing hard-to-reach or highly specialized populations in which participant identification and access may be challenging (47, 48). However, its use within professional football settings may also have resulted in a degree of network homogeneity, potentially restricting the diversity of perspectives. Additionally, existing professional relationships between participants may have influenced both access to the sample and the viewpoints expressed. These aspects should be considered when interpreting the findings and their transferability.

## 5 Conclusion

This study examined how professional tactical analysts conceptualise and apply the notion of playing styles in elite football. The findings indicate that analysts tend to rely on simplified and operational classifications, primarily focused on offensive behaviours such as possession-based, direct, and transitional play. Defensive behaviours were also identified, mainly in relation to the spatial organisation of teams when out of possession.

Beyond descriptive categorisation, the results highlight a clear conceptual distinction between playing style and game model, with the latter representing the context-dependent implementation of broader behavioural tendencies. From this perspective, playing styles should not be understood as fixed or discrete entities, but rather as adaptive and dynamic patterns that emerge from the interaction of multiple constraints, including the coach's philosophy, players' characteristics, level of competition, and contextual demands. In addition, a continuum between rigid and fluid expressions of playing styles was identified, reflecting teams' varying capacity either to impose their own approach or to adapt to the opponent.

This interpretation supports contemporary approaches that conceptualise team behaviour as a complex and emergent process and contributes to a more nuanced and ecologically valid understanding of tactical organisation in professional football.

## Data Availability

The datasets presented in this article are not readily available due to participant confidentiality and privacy considerations. A de-identified version of the dataset, with all potentially identifiable information removed, will be made available by the authors upon reasonable request. Requests to access the datasets should be directed to adrimaca@uax.es.

## References

[1] Francis JW Kyte J Bateman M. The role of the analyst: comparative analysis of applied performance analyst job advertisements in the UK and Ireland (2021–2022). Int J Perform Anal Sport. (2023) 24(2024):331–60. 10.1080/24748668.2023.2299178

[2] Aguado-Méndez RD González-Jurado JA Reina-Gómez Á Otero-Saborido FM. Perceptions of football analysts goal-scoring opportunity predictions: a qualitative case study. Front Psychol. (2021) 12:735167. 10.3389/fpsyg.2021.735167 34552540 PMC8450592

[3] Sarmento H Manuel Clemente F Araújo D Davids K McRobert A Figueiredo A. What performance analysts need to know about research trends in association football (2012–2016): a systematic review. Sports Med. (2018) 48:799–836. 10.1007/s40279-017-0836-6 29243038

[4] Fernandez-Navarro J Fradua L Zubillaga A Ford PR McRobert AP. Attacking and defensive styles of play in soccer: analysis of Spanish and English elite teams. J Sports Sci. (2016) 34(24):2195–204. 10.1080/02640414.2016.1169309 27052355

[5] Martín-Castellanos A Flores MR Solana DM Del Campo RL Garrosa FN Mon-López D. How do the football teams play in LaLiga? Analysis and comparison of playing styles according to the outcome. Int J Perform Anal Sport. (2024) 24(1):18–30. 10.1080/24748668.2023.2262813

[6] Lago-Peñas C Gómez-Ruano M Yang G. Styles of play in professional soccer: an approach of the Chinese soccer super league. Int J Perform Anal Sport. (2017) 17(6):1073–84. 10.1080/24748668.2018.1431857

[7] Zhou C Lago-Peñas C Lorenzo A Gómez MÁ. Long-Term trend analysis of playing styles in the Chinese soccer super league. J Hum Kinet. (2021) 79:237–47. 10.2478/hukin-2021-0077 34401003 PMC8336544

[8] Fernández-Navarro J. Analysis of Styles of Play in Soccer and Their Effectiveness. Granada: Universidad de Granada (2018).

[9] Hewitt A Greenham G Norton K. Game style in soccer: what is it and can we quantify it? Game style in soccer: what is it and can we quantify it? Int J Perform Anal Sport. (2016) 16(1):355–72. 10.1080/24748668.2016.11868892

[10] Plakias S Moustakidis S Kokkotis C Tsatalas T Papalexi M Plakias D. Identifying soccer Teams’ styles of play: a scoping and critical review. J Funct Morphol Kinesiol. (2023) 8(2):39. 10.3390/jfmk8020039 37092371 PMC10123610

[11] Plakias S Kasioura C Pamboris GM Kokkotis C Tsatalas T Moustakidis S. A grounded theory for professional soccer teams’ playing styles: towards a consensus. Int J Sports Sci Coach. (2024) 20(1):159–72. 10.1177/17479541241300605

[12] Plakias S Tsatalas T Betsios X Giakas G. A new era in soccer performance analysis research ? Insight - Sports Sci. (2025) 7(1):741. 10.18282/iss741

[13] Martín-Barrero A Martínez-Cabrera FI. Game models in soccer. From theoretical conception to practical design. Retos. (2019) 36:543–51. 10.47197/retos.v36i36.71021

[14] American Psychological Association. Ethical principles of psychologists and code of conduct. Am Psychol. (2002) 57(12):1060–73. 10.1037/0003-066X.57.12.1060 12613157

[15] Malterud K Siersma VD Guassora AD. Sample size in qualitative interview studies: guided by information power. Qual Health Res. (2016 Nov 1) 26(13):1753–60. 10.1177/1049732315617444 26613970

[16] Braun V Clarke V. Reflecting on reflexive thematic analysis. Qual Res Sport Exerc Health. (2019) 11(6):589–97. 10.1080/2159676X.2019.1628806

[17] Braun V Clarke V. One size fits all ? what counts as quality practice in (reflexive) thematic analysis ? Qual Res Psychol. (2021) 18:328–52. 10.1080/14780887.2020.1769238

[18] Braun V Clarke V. Using thematic analysis in psychology. Qual Res Psychol. (2006) 3:77–101. 10.1191/1478088706qp063oa

[19] Nowell LS Norris JM White DE Moules NJ. Thematic analysis: striving to meet the trustworthiness criteria. Int J Qual Methods. (2017) 16(1):1–13. 10.1177/1609406917733847

[20] Mayring P. Qualitative content analysis. In: Flick U von Kardorff E Steinke I, editors. A Companion to Qualitative Research. London: Sage (2004). p. 266–9.

[21] Plakias S. An integrative review of the game model in soccer: definition, misconceptions, and practical significance. Trends Sport Sci. (2023) 30(3):85–92. 10.23829/TSS.2023.30.3-1

[22] Sarmento H Pereira AA Matos N Campaniço J Anguera MT Leitão J. English Premier league, Spaińs la Liga and Italýs seriés A—what´s different? Int J Perform Anal Sport. (2013) 13(3):773–9. 10.1080/24748668.2013.11868688

[23] Pollard R Reep C Hartley S. The quantitative comparison of playing styles in soccer. In: Reilly T, Lees A, Davids K, Murphy WJ, editors. Science and Football. London: E. & F.N. Spon (1988). p. 309–15.

[24] Bangsbo J Peitersen B. Soccer Systems and Strategies. Champaign: Human Kinetics (2000).

[25] Perarnau M. La Evolución Táctica del Fútbol. 1st ed Barcelona: Córner (; 2021).

[26] Castelo JFF. Fútbol. Estructura y Dinámica del Juego. Barcelona: Inde (1999).

[27] Plakias S Tsatalas T Moustakidis S Giakas G Tsaopoulos D. Exploring the influence of playing styles on physical demands in professional football. Human Movement. (2023) 24(4):36–43. 10.5114/hm.2023.133919

[28] Eusebio P Prieto-González P Marcelino R. Decoding the complexities of transitions in football: a comprehensive narrative review. Ger J Exerc Sport Res. (2025) 55(3):332–42. 10.1007/s12662-024-00951-9

[29] Peng W Ribeiro J Wang M Gómez MÁ. Evolution of playing styles in UEFA European championships: trends from 2012 to 2024. Int J Perform Anal Sport. (2025):1–16. 10.1080/24748668.2025.2536954

[30] Ruan L Ge H Gómez MÁ Shen Y Gong B Cui Y. Analysis of defensive playing styles in the professional Chinese football super league. Sci Med Football. (2022) 7(3):279–87. 10.1080/24733938.2022.2099964 35796256

[31] Ruan L Ge H Shen Y Pu Z Zong S Cui Y. Quantifying the effectiveness of defensive playing styles in the Chinese football super league. Front Psychol. (2022) 13:899199. 10.3389/fpsyg.2022.899199 35719541 PMC9202555

[32] Freitas R Volossovitch A Almeida CH Vleck V. Elite-level defensive performance in football: a systematic review. Ger J Exerc Sport Res. (2023) 53(4):458–70. 10.1007/s12662-023-00900-y

[33] He Q Araújo D Davids K Kee YH Komar J. Functional adaptability in playing style: a key determinant of competitive football performance. Adapt Behav. (2023) 31(6):545–58. 10.1177/10597123231178942

[34] Plakias S Moustakidis S Kokkotis C Papalexi M Tsatalas T Giakas G. Identifying soccer players ‘ playing styles : a systematic review. J Funct Morphol Kinesiol. (2023) 8(3):104. 10.3390/jfmk8030104 37606399 PMC10443261

[35] Plakias S Moustakidis S Mitrotasios M Kokkotis C Tsatalas T Papalexi M. Analysis of playing styles in European football : insights from a visual mapping approach. J Phys Educ Sport. (2023) 23(6):1385–93. 10.7752/jpes.2023.06169

[36] Gasparetto T Shrainer A. The contribution of coaches in the playing style of football clubs. Int J Bus Perform Manag. (2025) 26(3):322–42. 10.1504/IJBPM.2025.145874

[37] Gama J Dias G Pereira M Mendes R Mendes RS Sarmento H. Network analysis to understand variability and patterns of individual and collective behaviour in professional football: a systematic review. Int J Perform Anal Sport. (2026):1–62. 10.1080/24748668.2026.2622759

[38] Xu Y Garcia JDC Sarmento H Zhong Y Gong B Yi Q. Application of social network analysis in football match analysis: a systematic review. Int J Sports Sci Coach. (2026) 21(1):548–68. 10.1177/17479541251377548

[39] González-Rodenas J Ferrandis J Moreno-Pérez V López-Del Campo R Resta R Del Coso J. Differences in playing style and technical performance according to the team ranking in the Spanish football LaLiga. A thirteen seasons study. PLoS One. (2023) 18(10):e0293095. 10.1371/journal.pone.0293095 37862370 PMC10588845

[40] Herrera-Diestra JL Echegoyen I Martínez JH Garrido D Busquets J Io FS. Pitch networks reveal organizational and spatial patterns of guardiola’s F.C. Barcelona. Chaos Solitons Fractals. (2020) 138:109934. 10.1016/J.CHAOS.2020.109934

[41] Satish K Faedo NI Ginesta X San Eugenio Vela JD. Possession football: the playing style that defined FC Barcelona’s success and the Spanish national football team’s golden generation. Managing Sport and Leisure. (2025):1–17. 10.1080/23750472.2025.2543794

[42] Gómez MÁ Mitrotasios M Armatas V Lago-Peñas C. Analysis of playing styles according to team quality and match location in Greek professional soccer. Int J Perform Anal Sport. (2018) 18(6):986–97. 10.1080/24748668.2018.1539382

[43] Michailidis Y Stafylidis A Vardakis L Kyranoudis A Mittas V Leftheroudis V. The running performance of elite youth football players in matches with a 1-4-3-3 formation in relation to their playing position. Appl Sci (Switzerland). (2025) 15(3984):3984. 10.3390/app15073984

[44] Aquino R Carling C Palucci Vieira LH Martins G Jabor G Machado J. Influence of situational variables, team formation, and playing position on match running performance and social network analysis in Brazilian professional soccer players. J Strength Cond Res. (2018) 34(3):808–17. 10.1519/jsc.0000000000002725 29985222

[45] Baptista J Travassos B Gonçalves B Mourao P Viana JL Sampaio J. Exploring the effects of playing formations on tactical behavior and external workload during football small-sided games. J Strength Cond Res. (2020) 34(7):1–20. 10.1519/JSC.0000000000002445 29337830

[46] Modric T Carling C Lago-Peñas C Sarmento H Veršić S Pajonková F. It is not (all) about running faster, opponent also plays; the effect of opposition team formation on running performance in professional soccer match-play. Int J Perform Anal Sport. (2025) 25(4):595–609. 10.1080/24748668.2024.2430099

[47] Noy C. Sampling knowledge: the hermeneutics of snowball sampling in qualitative research. Int J Soc Res Methodol. (2008) 11(4):327–44. 10.1080/13645570701401305

[48] Atkinson R Flint J. Accessing hidden and hard-to-reach populations: snowball research strategies. Soc Res Update. (2001) 33:1–4.

