# Supplementary material for: From playing styles to game model: deconstructing the paradigm through analysts' perspective
Source: Front Sports Act Living. 2026 Jul 24;8:1825139. doi: 10.3389/fspor.2026.1825139 (PMC13447262; doi:10.3389/fspor.2026.1825139)
Supplement: Supplementary file 1 [file Supplementaryfile1.docx]

**Annex: Interview model used**

**Warm-up questions:**

1. Your name is …
2. How old are you?
3. How many years have you been working as an analyst? And professionally?
4. Have you undertaken any studies related to football analysis?
5. How did you manage to access that role within the coaching staff?

**Topic 1: Analysis process**

- What is the analysis process of a team like?
- What do you take into account when analysing an opponent? How do you select those important aspects? Do you use the same analysis criteria when analysing your own team?
- Do you follow any kind of structure to carry out the analysis? If so, could you share it with us?
- Do you use any specific categorisation of teams in the analysis?

**Topic 2: Playing styles**

- How would you define what a playing style is? What playing styles could you name? What variables or information would you consider key to determine these playing styles?
- Which teams would you associate with each playing style? And in the Spanish league?
- How would you perceive that a team is playing in a certain style during the course of a match? What would you use to determine it? What information would you give to the rest of the coaching staff about that team?
- How would you determine that two teams play in a similar way?
- Is it possible to observe a relationship between playing styles and phases of play or game systems?
- How would you define your team’s playing style?

Thank you very much for everything, it has been a pleasure talking with you. If you would like to make any comments or add anything about the interview, this is the moment.
